# Intracellular Gold Nanoparticles Increase Neuronal Excitability and Aggravate Seizure Activity in the Mouse Brain

**DOI:** 10.1371/journal.pone.0091360

**Published:** 2014-03-13

**Authors:** Seungmoon Jung, Minji Bang, Byung Sun Kim, Sungmun Lee, Nicholas A. Kotov, Bongsoo Kim, Daejong Jeon

**Affiliations:** 1 Department of Bio and Brain Engineering, Korea Advanced Institute of Science and Technology (KAIST), Daejeon, Republic of Korea; 2 Department of Biomedical Engineering, Khalifa University of Science, Technology, and Research, Abu Dhabi, United Arab Emirates; 3 Department of Chemical Engineering, University of Michigan, Ann Arbor, Michigan, United States of America; 4 Department of Chemistry, Korea Advanced Institute of Science and Technology (KAIST), Daejeon, Republic of Korea; Georgia State University, United States of America

## Abstract

Due to their inert property, gold nanoparticles (AuNPs) have drawn considerable attention; their biological application has recently expanded to include nanomedicine and neuroscience. However, the effect of AuNPs on the bioelectrical properties of a single neuron remains unknown. Here we present the effect of AuNPs on a single neuron under physiological and pathological conditions *in vitro*. AuNPs were intracellularly applied to hippocampal CA1 neurons from the mouse brain. The electrophysiological property of CA1 neurons treated with 5- or 40-nm AuNPs was assessed using the whole-cell patch-clamp technique. Intracellular application of AuNPs increased both the number of action potentials (APs) and input resistance. The threshold and duration of APs and the after hyperpolarization (AHP) were decreased by the intracellular AuNPs. In addition, intracellular AuNPs elicited paroxysmal depolarizing shift-like firing patterns during sustained repetitive firings (SRF) induced by prolonged depolarization (10 sec). Furthermore, low Mg^2+^-induced epileptiform activity was aggravated by the intracellular AuNPs. In this study, we demonstrated that intracellular AuNPs alter the intrinsic properties of neurons toward increasing their excitability, and may have deleterious effects on neurons under pathological conditions, such as seizure. These results provide some considerable direction on application of AuNPs into central nervous system (CNS).

## Introduction

In the past decade, nanoparticles (NPs) have been used in biological and biomedical applications such as drug delivery, photothermal therapy, biosensing, and bioimaging [1], [2]. NPs are typically transferred into the cells by endocytosis [3]. NPs can be used for delivery of genes or drugs into the cytosol and subcellular organelles, including the nucleus [4]. In particular, intracellular dynamics could be monitored by intracellular delivery of nano-sized contrast agents, and targeting nanomedicine into subcellular organelles could vastly improve the efficacy of therapeutic regimens such as proapoptotic drugs, lysosomal enzymes, gene therapy, and photodynamic therapy [4], [5]. However, many toxic effects of NPs on various types of cells have been widely known [6], [7].

Gold NPs (AuNPs) are of particular interest due to their excellent stability and various biocompatibility properties, including nontoxicity, non-immunogenicity, and high tissue permeability without hampering cell functionality [8], [9]. The distinct properties of AuNPs suggest their potential for the delivery of therapeutic substances—such as drugs or small nucleotides—into the brain for treatment of various neurological diseases or disorders [10]. Although novel AuNPs have been developed as carriers for delivery of therapeutic substances to neuronal cells across the blood-brain barrier (BBB) [11], [12], the effect of AuNPs on the electrophysiological activity of neurons has not been investigated. Recently, several NPs, such as silver (Ag), copper oxide (CuO), zinc oxide (ZnO), and tungsten carbide (WC), were reported to alter some properties of ion channels and neuronal excitability [13]–[19]. Although it remained unclear that these NPs affected inside or outside of neuronal cells, these works suggested some deleterious or toxic effects of NPs on bioelectrical properties of neurons in the brain.

The neuron is a cellular unit of the nervous system. Neuronal activity is represented by changes in membrane potential, such as action potentials (APs), and the related synaptic transmission by means of neurotransmitters, which is associated with receiving, integrating, and transmitting information in the brain [20]. The intrinsic properties of ion channels in neurons determine or regulate neuronal activity [21], and alterations in neuronal activity could impact both physiological and pathophysiological conditions. For instance, abnormal neuronal activity or imbalanced excitation/inhibition is associated with several neurological diseases, such as epilepsy [22], [23]. Therefore, for biological and biomedical applications of AuNPs as intracellular carriers of specific molecules (e.g., a drug, antibody, or oligonucleotide) to the brain, a thorough understanding of the interplay between intracellular AuNPs and neuronal intrinsic properties is necessary [24], [25].

Patch-clamp recording is an approach to measure bioelectrical properties of the cells, and is especially useful in the study of excitable cells such as neurons [26]–[28]. One of advantages in whole-cell patch-clamp recordings is to deliver substances directly inside of a living cell through a glass micropipette. Thus, it is possible to investigate the intracellular effect of substances on the bioelectrical properties of a single neuron by whole-cell patch-clamp recordings. In this study, we measured the electrophysiological properties of neurons using the whole-cell patch-clamp technique after delivering AuNPs intracellularly into hippocampal CA1 neurons from the mouse brain. We also investigated the effect of intracellular AuNPs on two *in vitro* seizure models (prolonged sustained repetitive firings and low Mg^2+^-induced epileptiform burst discharges). Herein is the first report of the effects of AuNPs on the bioelectrical properties of a single neuron under physiological and pathological conditions.

## Materials and Methods

### Ethics Statement

Animal care and handling were conducted according to the guidelines approved by the Institutional Animal Care and Use Committee (Approval Number: KA2013-33) of the Korea Advanced Institute of Science and Technology (KAIST). All efforts were made to minimize suffering.

### Animals

Young male *C57BL/6* mice (4–5 weeks old) were used in the present study. Four mice were housed as a group under a 12-hr light/dark cycle with free access to food and water.

### Nanoparticles and application to brain slice

AuNPs (OD 1, stabilized suspension in citrate buffer, negatively charged) of 5- and 40-nm diameter were purchased from Sigma (St. Louis, MO, USA). Fluorophore-labeled (maximum absorbance: 600 nm) spherical AuNPs (40 nm, methyl conjugated) were purchased from Nanopartz Inc. (Loveland, CO, USA). AuNPs were stored at 4°C before use. For intracellular application of AuNPs, the AuNPs were diluted with an intrapipette solution (approximately 1.1×10^11^ NPs/mL for 5-nm AuNPs, and 1.44×10^8^ NPs/mL for 40-nm AuNPs). Electrophysiological recordings with citrate-suspended, non-fluorescent AuNPs were performed 10 min after cell rupture.

### Brain slice preparation and patch-clamp recordings

Preparation of hippocampal slices and the whole-cell patch-clamp recording method have been described previously [29], [30]. Fully anesthetized mice were decapitated and the horizontal hippocampal slices (310 μm) were prepared in oxygenated (95% O_2_, 5% CO_2_), cold, ACSF (124 mM NaCl, 3.0 mM KCl, 1.23 mM NaH_2_PO_4_, 2.2 mM CaCl_2_, 1.2 mM MgCl_2_, 26 mM NaHCO_3_, and 10 mM glucose, pH 7.4). After 1 hr recovery, brain slices were incubated in ACSF and whole-cell recordings were obtained from hippocampal CA1 neurons at 31 °C using glass pipette electrodes (3–6 MΩ). To measure APs and miniature excitatory postsynaptic currents (mEPSCs), glass pipettes were filled with an internal solution (135 mM K-gluconate, 5 mM KCl, 2 mM MgCl_2_, 5 mM EGTA, 10 mM HEPES, 0.5 mM CaCl_2_, 5 mM Mg-ATP, and 0.3 mM Na-GTP) which was buffered to pH 7.4 with KOH. APs were triggered by a step-current injection (30 pA steps) from −150 pA to +150 pA in current-clamp mode for 1 sec. The numbers, threshold, and latency of APs evoked by the injected currents in AuNPs-treated and untreated neurons were analyzed. The duration of the first AP was measured at half amplitude above the threshold. The after hyperpolarization (AHP) amplitude was isolated from the first AP. Spontaneous firings in CA1 neurons were measured at −50 mV. After the neurons had been voltage-clamped at −60 mV, the mEPSC experiment was performed in the presence of 1 μM tetrodotoxin (TTX), 10 μM bicuculline (GABA_A_ receptor antagonist), and 5 μM CGP 55845 (GABA_B_ receptor antagonist). For the prolonged sustained repetitive firing (SRF) experiment, 80–90-pA currents were injected into the cell under current-clamp configuration for 10 sec [31]. The SRF experiment was conducted more than two times in each of cell. To precipitate epileptiform burst discharges, brain slices were incubated in low-Mg^2+^/high-K^+^ ACSF containing the following (in mM): 124 mM NaCl, 5.0 mM KCl, 1.23 mM NaH_2_PO_4_, 2.2 mM CaCl_2_, 26 mM NaHCO_3_, and 10 mM glucose [32]. After 1 hr incubation, epileptiform activity was measured with the same low-Mg^2+^/high-K^+^ ACSF. The low-Mg^2+^/high-K^+^ ACSF elicited bursts of spikes, and the number of bursts showing more than three spikes was analyzed for 3 min. In all the patch-clamp recordings, large cells in hippocampal CA1 region were visually chosen, and pyramidal neurons and interneurons were identified on the basis of their distinctive intrinsic membrane properties including firing patterns as described previously [33]–[36]. Cells showing intrinsic membrane properties of interneurons were removed from analysis. Patch-clamp recordings were performed using a MultiClamp 700 B amplifier and a Digidata1440 (Axon instruments), and the acquired data were analyzed using the pCLAMP version 10.2 (Axon Instruments) and Mini-Analysis Program (Synaptosoft).

### Statistical analysis

All data are presented as means ± standard error of the mean (SEM). Statistical analyses were conducted using the SPSS software (SPSS, Chicago, IL, USA) and R (Software Foundation, Boston, MA, USA). Data were analyzed by analysis of variance (ANOVA) followed by *post hoc* comparisons. Student's *t*-test was used to identify main effects. A *p*-value <0.05 was considered to indicate statistical significance.

## Results

### Altered passive electrical properties by an intracellular application of AuNPs

**Figure 1:**
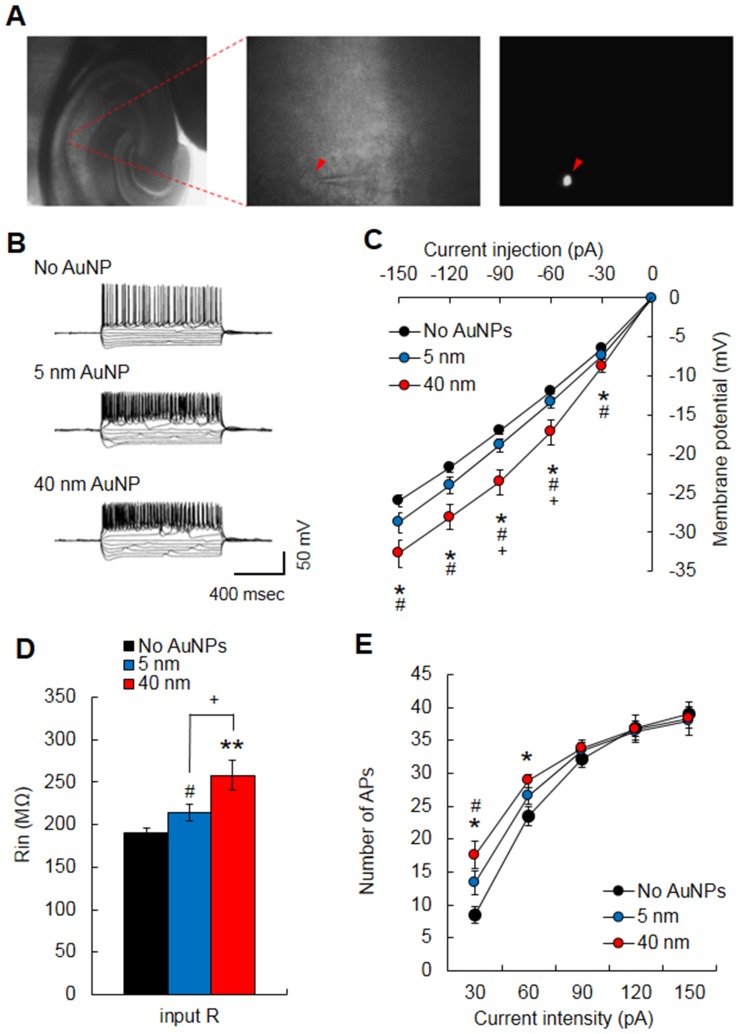
Effects of intracellular treatment with 5- or 40-nm AuNPs on the passive electrical properties of hippocampal CA1 neurons from a mouse hippocampal slice. (A) DIC images of a brain slice (left, 50×) and hippocampal CA1 layer (middle, 630×), and a fluorescence image of CA1 neurons (right, 630×) loaded with fluorophore-conjugated AuNPs through a patch pipette (middle) after breaking the gigaohm seal. The fluorescence signal indicates the infusion of AuNPs into the cell. (B) Representative traces of membrane potential changes and APs elicited by step-current injections for 1 sec from AuNP-treated and untreated (no AuNPs) hippocampal CA1 neurons. (C) AuNPs of both sizes considerably increased the changes in membrane potential. (D) Input resistance was significantly increased by AuNPs of both sizes. (E) The 5- or 40-nm AuNPs increased the number of APs substantially at low current intensity (at 30- or 60-pA depolarizing current injection). *p<0.05, **p<0.01, Student's *t*-test, No AuNPs *vs*. 40-nm AuNPs; ^#^ *p*<0.05, Student's *t*-test, No AuNPs *vs*. 5-nm AuNPs; ^+^ *p*<0.05, Student's *t*-test, 5-nm AuNPs *vs*. 40-nm AuNPs.

To investigate the effect of AuNPs on the electrophysiological properties of a single neuron, AuNPs were added to the hippocampal CA1 neurons from a mouse brain slice using a glass micropipette after being mixed with an intrapipette solution. We first verified the intracellular distribution of AuNPs by using fluorophore-conjugated 40-nm AuNPs, which showed fluorescent signals inside of neurons (Figure 1A). The spherical cell shape was clearly displayed by the fluorescent signals, which indicates that the AuNPs were evenly and broadly distributed throughout the cell body and membrane (Figure 1A, right). Then, non-fluorescent AuNPs of 5- and 40-nm diameter were used for the intracellular application throughout the experiments. Passive membrane properties, such as input resistance, threshold potential for AP generation, and firing frequencies against the amplitude of injected currents, were measured using a current-clamp configuration. APs were generated by current injections with 30 pA steps (Figure 1B). Current-voltage relationships were obtained from values measured at the middle (500 msec) of hyperpolarizing pulses (Figure 1C). The plotted relationship curve shifted downward in both 5- (*n = *18) and 40-nm (*n = *12) AuNP-treated neurons compared to non-treated CA1 neurons (*n = *29) (F(2,56) = 45.91, *p<*0.001, two-way ANOVA), and changed more with the 40-nm AuNPs than with 5-nm AuNPs (F(1,28) = 22.32, *p<*0.001, two-way ANOVA) (Figure 1C). There was also a significant difference in the input resistance between AuNP-treated and non-treated neurons (F(2,56) = 11.25, *p<*0.05, one-way ANOVA). The input resistance was increased in both 5- (214.22±9.94 MΩ, *p<*0.05, Student's *t*-test) and 40-nm (258.1±17.45 MΩ, *p<*0.001, Student's *t*-test) AuNP-treated neurons compared to non-treated CA1 neurons (190.87±5.59 MΩ) (Figure 1D), and changed more with the 40-nm AuNPs than with 5-nm AuNPs (*p<*0.05, Student's *t*-test). As a measure of neuronal excitability, we plotted firing frequencies against the intensity of injected currents. Increased number of spikes was observed at low intensities of current injection. Both 5- (13.33±1.76) and 40-nm AuNP-treated neurons (17.58±2.04) showed significantly more spikes at +30 pA injections than the non-treated neurons (8.51±1.22) (*p<*0.05, Student's *t*-test) (Figure 1E). The 40-nm (28.83±0.99), but not 5-nm, AuNP-treated neurons also showed significantly more spikes at +60 pA injections than the non-treated neurons (23.51±1.43) (*p<*0.05, Student's *t*-test). Collectively, these results suggest that intracellular AuNPs alter the basic bioelectrical properties of hippocampal CA1 neurons.

### The properties of AP are altered by an intracellular application of AuNPs

**Figure 2:**
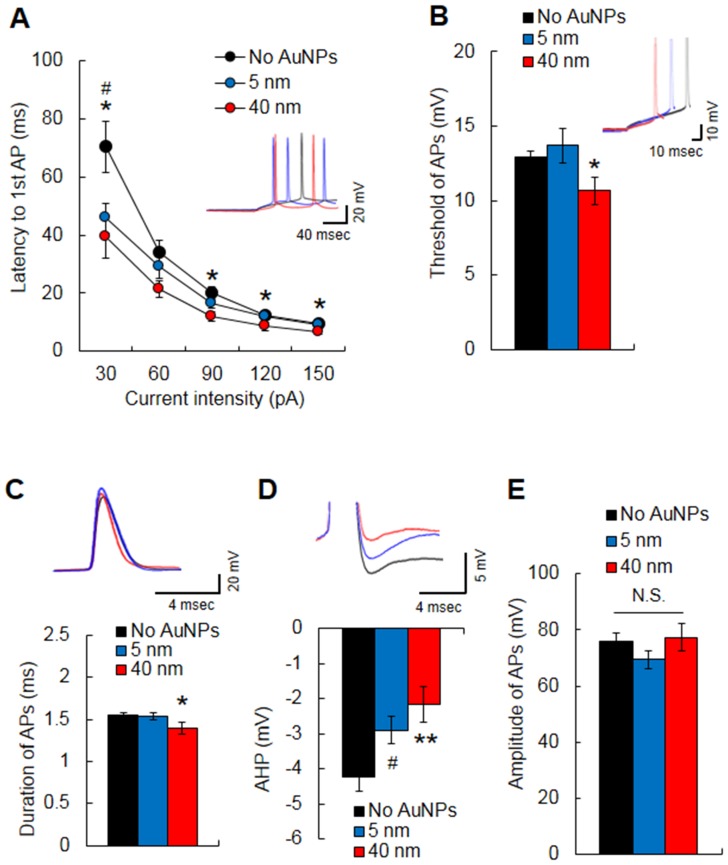
Effects of intracellular treatment with 5- or 40-nm AuNPs on AP properties in hippocampal CA1 neurons. (A) AuNPs of both sizes significantly decreased the latency to the first AP. The 40-nm AuNPs significantly reduced the AP threshold (B) and AP duration (C). (D) AuNPs of both sizes significantly increased AHP. (E) AuNPs did not affect the AP amplitude. *p<0.05, **p<0.01, Student's *t*-test, No AuNPs *vs*. 40-nm AuNPs; ^#^ *p*<0.05, Student's *t*-test, No AuNPs *vs*. 5-nm AuNPs; N.S., no significance.

Regarding neuronal excitability, we further analyzed the properties of APs in CA1 neurons treated with AuNPs intracellularly. The latency to the first spikes at injection of a current of each intensity was noticeably decreased after the intracellular application of 40-nm AuNP-treated neurons (F(1,41) = 15.70, *p<*0.001, two-way ANOVA) (Figure 2A). The 5-nm AuNP-treated neurons showed significant change at only 30 pA injection (*p<*0.05, Student's *t*-test). In addition, 40-nm AuNPs decreased the threshold amplitude of the first AP generation (non-treatment, *n = *23, 12.89±0.43 mV; 40 nm, *n = *12, 10.69±0.91 mV, *p<*0.05, Student's *t*-test) (Figure 2B) and shortened the duration of the first AP (Figure 2C) compared with non-treatment (non-treatment, 1.54±0.03 ms; 40 nm, 1.39±0.07 ms, *p<*0.05, Student's *t*-test). However, 5-nm AuNPs did not affect the threshold amplitude (13.7±1.18 mV) and duration (1.53±0.04 ms) of the first AP generation. The AHP was significantly reduced with both AuNP sizes (5 nm, −2.9±0.39 mV; 40 nm, −2.16±0.51 mV) compared with non-treatment (−4.24±0.39 mV) (F(2,50) = 6.10, *p*<0.005) (Figure 2D), and there was no significant difference in the AHP amplitude between the 5-nm and 40-nm AuNP-treated neurons. No difference in the amplitude of APs was observed between the AuNP-treated and non-treated neurons (Figure 2E). These results demonstrate that intracellular AuNPs may lead CA1 neurons to become more excitable. Considering the alteration in basic bioelectrical properties, not only the ion channels active in the subthreshold range but also the channels activated during APs are subjected to alterations in CA1 neurons treated intracellularly with AuNP.

### Effects on spontaneous firings and mEPSC of intracellular application of AuNPs

**Figure 3:**
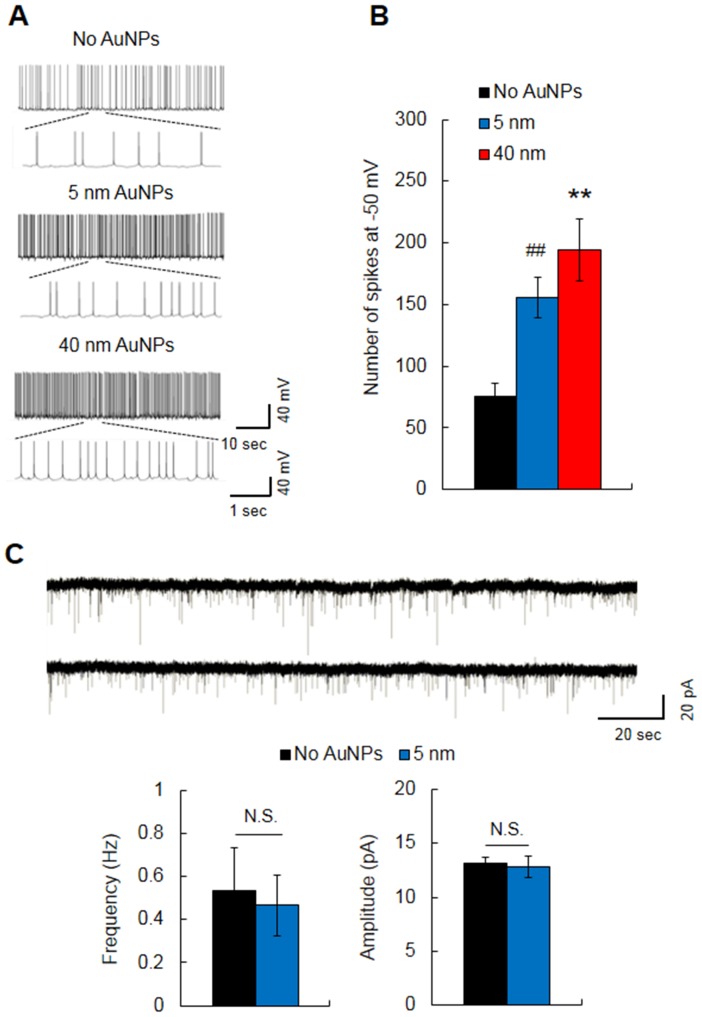
Effects of intracellular treatment with 5- or 40-nm AuNPs on spontaneous firing and excitatory synaptic transmission. (A) The representative traces of spontaneous firing from AuNP-treated and non-treated (No AuNPs) hippocampal CA1 neurons. (B) AuNPs of both sizes significantly increased the rate of spontaneous firing. (C) Neurons treated with 5-nm AuNP showed similar mEPSC frequency and amplitude to non-treated neurons. **p<0.01, Student's *t*-test, No AuNPs *vs*. 40-nm AuNPs; ^##^ *p*<0.01, Student's *t*-test, No AuNPs *vs*. 5-nm AuNPs; N.S., no significance.

Next, we examined whether spontaneous firings in CA1 neurons are affected by intracellular treatment with AuNPs. Spontaneous APs were measured at −50 mV in current-clamp mode (Figure 3A). The 5- (*n* = 7, 155.86±16.52) and 40-nm (*n = *11, 194.55±25.29) AuNP-treated neurons displayed more than twice the number of spikes than non-treated neurons (*n = *13, 75.46±10.73) (F(2,28) = 12.36, *p<*0.001, one-way ANOVA) (Figure 3B). To determine whether the AuNPs affected excitatory synaptic transmission—which can alter spontaneous firing—we measured mEPSC in 5-nm AuNP-treated neurons. There was little difference in the frequency (Figure 3C, Left) and amplitude (Figure 3C, Right) of mEPSC between AuNP-treated (*n* = 6) and non-treated neurons (*n* = 7). Thus, the increased firing rate may be due to the altered intrinsic properties of CA1 neurons themselves rather than increased excitatory synaptic transmission. These results indicate that intracellular treatment with AuNPs enhanced the excitability of CA1 neurons.

### Effects of AuNPs on prolonged depolarization and low Mg^2+^-induced epileptiform discharges

**Figure 4:**
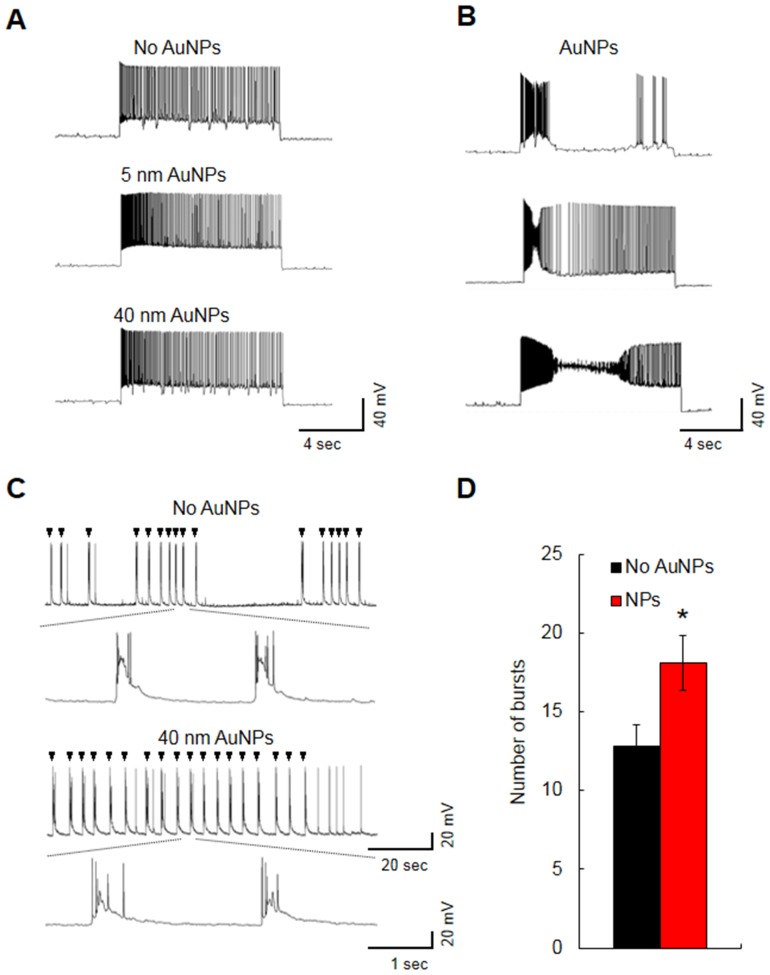
Effects of intracellular treatment with 5- or 40-nm AuNPs on seizure models. (A, B) Prolonged SRF experiment. (A) The representative traces of repetitive firings elicited by long (10 sec) depolarizing current pulses. (B) PDS-like spikes, an epiletiform activity, were observed from a portion of AuNP-treated hippocampal CA1 neurons. (C) The representative traces of low Mg^2+^-induced bursts of spikes. Arrow head indicates a burst of spikes. (D) Intracellular 40-nm AuNPs increased the number of bursts. Average number of bursts per min were presented. *p<0.05, Student's *t*-test; N.S., no significance.

Increased excitability can lead to neurological diseases, such as epilepsy. Thus, we investigated the effects of intracellular treatment with AuNPs on two *in vitro* seizure models (prolonged SRF and low Mg^2+^-induced epileptiform burst discharges) [31], [32]. In prolonged SRF experiment, about 70% of AuNP-treated CA1 neurons showed a similar firing pattern to non-treated neurons (5-nm AuNP-treated, *n = *8/11; 40-nm AuNP-treated, *n = *10/14; untreated CA1 neurons *n = *18) (Figure 4A). However, approximately 30% neurons (7 of 25 neurons) repetitively displayed abnormal eccentric firing behaviors during the long depolarization (Figure 4B). Interestingly, the abnormal firings induced by intracellular AuNPs look very similar to a paroxysmal depolarizing shift (PDS), a cellular manifestation of epileptic seizure caused by excessive ionic currents, an imbalance in the ionic distributions, or dysfunctions of ion channels such as Na^+^ or Ca^2+^ channels [37]. The abnormal firing patterns were never observed in non-treated CA1 neurons. With regard to low-Mg^2+^-induced epileptiform activity, AuNP-treated (40 nm, *n = *12, 18.08±1.74) neurons showed significantly increased number of bursts compared to untreated CA1 neurons (*n = *8, 12.82±1.31) (*p<*0.05, Student's *t*-test) (Figure 4C and 4D). Taken together, these results indicate that the increased excitability of AuNP-treated neurons is likely to result in the hyperexcitability implicated in pathological conditions such as seizure.

## Discussion

The unique physical properties of NPs, including their small size and ability to cross the BBB, are potential advantages for delivering drugs, genes and other small molecules into the brain [38], [39]. AuNPs are widely used in biotechnology because of their excellent biocompatibility. However, their physiological influence on neuronal cells has to date not been investigated extensively. In this study, we examined the effects of intracellular application of AuNPs on the activity of a single neuron using patch-clamp recording. Intracellular treatment with AuNPs increased the input resistance and number of spikes, decreased the latency and threshold of AP generation, reduced the AHP, and enhanced spontaneous firing rate in hippocampal CA1 neurons. Furthermore, the AuNPs elicited PDS-like epileptiform activity in a portion of neurons during a long depolarization and increased the number of bursts in low Mg^2+^-induced *in vitro* seizure model. These results demonstrate that intracellular AuNPs increase the excitability of neurons and aggravate the irritability of neurons in pathological conditions, such as seizure. In this regard, our data suggest that the delivery of AuNPs as vehicles to carry therapeutic agents into CNS should be carefully considered despite their well-known advantage and biocompatibility.

Many studies have reported that NPs including AuNPs have toxicity involving cell damage or death [20], [40]–[42]. The kinetics of bioactive molecules such as a drug or protein, are likely to differ considerably intra- and extracellularly [43], [44]. Furthermore, an increasing number of studies on drug delivery to the brain has focused on intracellular labeling of proteins to enhance the effectiveness of the cargo molecules in nanomedicine [4], [5]. Recently, application of several NPs, such as Ag, CuO, ZnO, and WC, has been reported to alter the properties of ion channels and excitability in neurons of the brain [13]–[19]. Ag-NPs have been shown to enhance glutamatergic synaptic transmission and the neuronal firing rate in rat hippocampal slices [15], [18]. CuO- and ZnO-NPs were suggested to affect sodium or potassium currents and enhance the excitability of acutely isolated rat hippocampal CA3 neurons [13], [14]. In a study of WC-NPs, the NPs reduced the number of APs [19]. However, these studies did not provide the direct evidence on the pathophysiological effects of the NPs on the neuronal cells. The present study showed that intracellular AuNPs led to abnormal firing patterns and aggravated epileptic activity under pathological conditions. Thus, our study suggest a possibility that intracellular AuNPs can cause or worsen neuronal dysfunction or damage in the brain.

In neurons, ions diffuse along the electrochemical gradient, and the passive diffusion of ions through open channels create currents. These currents can then alter neuronal membrane potentials. Ion channels in the plasma membrane are the primary determinant of the bioelectrical properties of neurons in the brain. Various modes of the effects of AuNPs on ionic flow could be hypothesized. Although we could not identify the ion channel affected by AuNPs in this study, AuNPs might interact with Na^+^ and K^+^ channels. Voltage-gated Na^+^ and K^+^ channels play a critical role in the generation or shaping of APs [45], and Ca^2+^-activated K^+^ channels together with voltage-gated K^+^ channels regulate AHP or spike frequency in neurons [46], [47]. In our study, the amplitude of AP was not altered by the AuNPs; therefore, the AuNPs were unlikely to significantly affect the voltage-gated Na^+^ channels. However, modulation of the kinetics of voltage-gated Na^+^ channels by the AuNPs cannot be ruled out [46]. K^+^ channels including Ca^2+^-activated K^+^ channels appear to have the capacity to interact with AuNPs. For instance, pharmacological blocks or genetic mutations of K_v_12.2, a slowly activating voltage-gated K^+^ channel, led to increased input resistance and number of APs in compliance with small electric stimuli [48]. Pharmacological block of Ca^2+^-activated K^+^ channels also cause increased excitability by reducing AHP and increasing the spike frequency in neurons [49]. The intracellular interaction of AuNPs with specific modules of ion channels may be regarded as another aspect of the effects of AuNPs. Voltage-gated ion channels are generally composed of two main parts, the pore-forming transmembrane domains (including voltage-sensing modules), and cytoplasmic loops [50]. Recent studies showed that the pores of ion channels such as hERG and nicotinic acetylcholine receptor (nAChRs) can be directly clogged with ultra-small AuNPs (∼1.4-nm diameter) and impede the movement of ions through the channel pore [51], [52]. However, the estimated pore sizes of Na^+^ and K^+^ channels are either below or approximately 10 Å in diameter [53], [54]; therefore, the AuNPs used in our study were too large to physically internalize within the channels' pore. Further studies should identify the ion channels affected by the AuNPs by measuring the ionic currents within channels and examine the mechanisms underlying the interaction of the AuNPs with the ion channels.

In conclusion, we demonstrated that intracellular AuNPs caused hippocampal CA1 neurons to be more excitable in terms of generating more APs, which might result from the reduced threshold and duration of AP, increased input resistance, and reduced AHP amplitude. We also examined the effects of AuNPs on *in vitro* pathological conditions. Occasionally, intracellular AuNPs elicited eccentric firing patterns during a long-depolarization, and aggravated epileptiform activity in a seizure model *in vitro*. Thus, the intracellular AuNPs might lead to disturbances in neuronal functions and hyperexcitability in pathological conditions such as seizure. Our results provide valuable information for the use of AuNPs in nanomedicine as an intracellular drug-delivery system.
